# A global gas flaring black carbon emission rate dataset from 1994 to 2012

**DOI:** 10.1038/sdata.2016.104

**Published:** 2016-11-22

**Authors:** Kan Huang, Joshua S. Fu

**Affiliations:** 1 Center for Atmospheric Chemistry Study, Shanghai Key Laboratory of Atmospheric Particle Pollution and Prevention (LAP^3^), Department of Environmental Science and Engineering, Fudan University, Shanghai 200433, China; 2 Department of Civil and Environmental Engineering, The University of Tennessee, Knoxville, Tennessee 37996, USA; 3 Climate Change Science Institute and Computer Science and Mathematics Division, Oak Ridge National Laboratory, Oak Ridge, Tennessee 37831, USA

**Keywords:** Atmospheric chemistry, Environmental chemistry

## Abstract

Global flaring of associated petroleum gas is a potential emission source of particulate matters (PM) and could be notable in some specific regions that are in urgent need of mitigation. PM emitted from gas flaring is mainly in the form of black carbon (BC), which is a strong short-lived climate forcer. However, BC from gas flaring has been neglected in most global/regional emission inventories and is rarely considered in climate modeling. Here we present a global gas flaring BC emission rate dataset for the period 1994–2012 in a machine-readable format. We develop a region-dependent gas flaring BC emission factor database based on the chemical compositions of associated petroleum gas at various oil fields. Gas flaring BC emission rates are estimated using this emission factor database and flaring volumes retrieved from satellite imagery. Evaluation using a chemical transport model suggests that consideration of gas flaring emissions can improve model performance. This dataset will benefit and inform a broad range of research topics, e.g., carbon budget, air quality/climate modeling, and environmental/human exposure.

## Background & Summary

Flaring is a common method of disposing of gaseous and liquid hydrocarbons through combustion at oil/gas production and processing sites due to a lack of pipelines and other gas transportation infrastructure, as well as for protection against the dangers of over-pressurizing industrial plant equipment. According to the latest estimates from satellite data, the total flared gas volume was estimated at 143 billion cubic meters (BCM) in 2012, accounting for 3.5% of overall global production^1^. Annual economic losses related to gas flaring are estimated to be more than 5 and 11 billion dollars for Russia^2^ and Nigeria^3^, which are the top two biggest contributors to gas flaring volumes in the world. Gas flaring has had a number of environmental and socioeconomic impacts, including changes in microclimate (e.g., temperature), reduction of bacterial and fungi, damage to economically valuable plant species^4^, oil contamination of soils and degradation of adjacent ecosystems^5^, increased risk of acid deposition^6^, alteration to the behavior of night-migrating birds^7^, increased risks of abortion, stillbirth, and mortality in domestic animals^8^, and reduced life expectancy^9^.

Atmospheric pollutants from gas flaring include CO_2_, CO, CH_4_, NOx, N_2_O, H_2_S, hydrocarbons, PM, etc. Of the emitted gaseous species, CH_4_ and N_2_O are both greenhouse gases with much higher global warming potential than CO_2_. In addition, the associated gas containing H_2_S (sour gas) can be very toxic. PM from gas flaring mainly takes the form of soot or black carbon^10^. Black carbon (BC) is thought to be second to carbon dioxide in terms of global warming^11^. It directly warms the atmosphere by absorbing sunlight and also indirectly, reducing surface albedo via deposition on ice and snow covers. In contrast to greenhouse gases with long lifetimes, BC usually stays in the atmosphere for days to weeks, and thus is considered to be a short-lived climate forcer (SLCF). In this light, control of black carbon particles by emission reduction measures would have immediate benefits for air quality and global warming^12^.

Compared to the traditional emission sectors, BC emission measurements from gas flaring have been relatively little studied. By using an advanced optical technique sky-LOSA (Line-Of-Sight Attenuation), the emission factor measured in the gas flaring field of Uzbekistan^13^ and Mexico^14^ was determined to be 2±0.66 and 0.067±0.02 g s^−1^, respectively. Nine flight measurements in March 2014 over the Bakken oil-producing region of North Dakota estimated a flaring BC emission factor of 0.13±0.36 g m^−3^ and an upper bound value of 0.28 g m^−3^ based on black carbon mass (SP2: Single Particle Soot Photometer) and absorption measurements (PSAP: Particle Soot Absorption Photometer), respectively^15^. Later, a flight campaign in May 2014 over the same region using SP2 estimated the upper limit of the flaring BC emission factor to be 0.57±0.14 g m^−3^ (ref. 16). This indicates substantial differences in emission factors among different fields and even between flares at the same field. The ECLIPSE (Evaluating the CLimate and Air Quality ImPacts of Short-livEd Pollutants) emission inventory (before Version 5a) used a gas flaring BC emission factor of 1.6 g m^−3^ for most of the regions in the world^17^. ECLIPSE V5a is starting to consider the differences of associated petroleum gas (APG) composition among some regions. By improving the emission factors, future policy decisions could be made more applicable in various aspects, e.g., the assessment of the magnitudes of climate benefits from gas flaring BC emission reduction in the cryosphere regions conducted by a joint effort of World Bank and the International Cryosphere Climate Initiative^18^, and more accurate estimation of temperature response to gas flaring from Russia in the Arctic^19^.

The petroleum and other liquid fuel production from OPEC and non-OPEC sources are predicted to increase continuously by 23 million b/d, from 76 million b/d in 2012 to 100 million b/d in 2040 (ref. 20). Although the ‘Zero Routine Flaring by 2030’ initiative launched by the World Bank attracted nine countries as of 2015, some major flaring countries still have not participated, as this target is hard for them to achieve. In addition, countries such as the USA and Canada have rapidly contributed to an increase in flaring since 2006 (ref. 21). Hence, understanding the past and current status of gas flaring BC emissions becomes imperative for making sound solutions to eliminating routine flaring. In this study, we present the procedures of an open-access archive of temporally comparable, high-resolution datasets of gridded gas flaring BC emissions from 1994 to 2012 by developing region-dependent gas flaring emission factors and usage of satellite imagery data. The data products are validated with the numerical simulation method.

## Methods

### Region-dependent gas flaring black carbon emission factors

**Figure 1:**
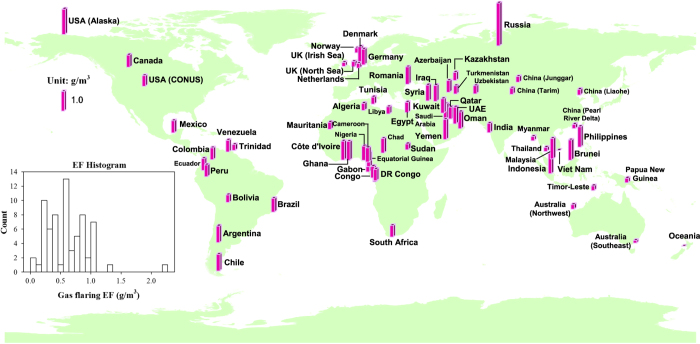
The global map of the mean values of EF_*flare,BC*_ for various gas flaring regions. A histogram of EF_*flare,BC*_ is shown inside the bottom left corner of the figure. Abbreviations of some regions are defined as: USA—United States of America; CONUS—Conterminous United States; UK—United Kingdom of Great Britain and Northern Ireland; DR Congo—Congo (Democratic Republic of the); UAE—United Arab Emirates.

As pointed out in various studies^13,14,17,22^, the gas flaring BC emission factors could vary significantly among different oil and gas fields. McEwen and Johnson^10^ established the first empirical relationship between BC emission factors and volumetric fuel heating values for a range of conditions by imitating flares at the laboratory scale. They derived a linear regression equation as follows:

(1)EFflare,BC=0.0578×HVAPG–2.09(correlation:R2=0.85),

where EF_*flare,BC*_ and HV_*APG*_ represent the gas flaring BC emission factor and the volumetric weighted heating value of APG, respectively. According to Equation (1), EF_*flare,BC*_ present a strong linear relationship with HV_*APG*_. Hence, the chemical composition of APG is one of the parameters for determining the gas flaring BC emission factor. It has to be noted that other factors such as operating practices could also greatly affect the gas flaring emission factors. However, the regional variability of operating practices has been hard to gather and translated to quantitative uncertainties. Hence, in this study, we mainly focus on the impact of APG compositions on the variability of gas flaring BC emission factors. By extensively collecting APG information from literatures, reports and other resources^15,16,22–60^, we establish a global database of APG composition, HV_*APG*_, and EF_*flare,BC*_ for the first time (Data Citation 1). It should be noted that APG information is not available for some countries, so we have used APG information from adjacent countries for the substitution. For instance, no information of APG composition is available for Oman, so we use that of United Arab Emirates to represent Oman. Other examples are indicated in the ‘Remark’ column in (Data Citation 1). APG information is fairly limited in most countries or regions. Field sampling with laboratory measurements from more oil fields in the future are desired to generate a more comprehensive database with stronger spatial variability of APG compositions. Heating values of APG for each region are calculated on the weighted volume basis. Finally, the values of EF_*flare,BC*_ are calculated according to Equation (1). It should be noted that EF_*flare,BC*_ are always presented in certain ranges. The uncertainties of EF_*flare,BC*_ are considered from two aspects; first, as for a specific region, the APG compositions are variable among its different oil and gas fields. Even for the same oil and gas field, APG compositions could vary by multiple measurements due to changed conditions such as temperature, pressure, etc. For example, the APG data in the North Sea off the United Kingdom are taken from 13 offshore fields. Secondly, McEwen and Johnson^10^ indicated the regression algorithm (Equation 1) is associated with uncertainties of <21%. In this study, we use the across-the-board value of 20% applied to the uncertainty calculation of EF_*flare,BC*_ for all the regions. It should be especially noted that the uncertainty analysis introduced in this study only accounts for the two aspects referred above, i.e., the variability of APG compositions and experimental errors. Other factors such as operating practices are not considered for uncertainty analysis in this study as this information on a global scale is rare and not quality-assured. The upper limit, lower limit, and mean value of EF_*flare,BC*_ are recorded in (Data Citation 1). Figure 1 presents a global map of the mean values of EF_*flare,BC*_ for gas flaring regions identified by the NOAA DMSP nighttime light products (see flaring regions in the following section). The histogram of EF_*flare,BC*_ (inner plot in the bottom left corner of Fig. 1) indicates that EF_*flare,BC*_ varies across a wide range, from a minimum of almost zero to a maximum of 2.27 g m^−3^ (Russia). Around 37% of the regions have EF_*flare,BC*_ of 0.2–0.5 g m^−3^, and around 56% of the regions have EF_*flare,BC*_ higher than 0.6 g m^−3^.

### Spatial distribution of gas flaring BC emission rates

Elvidge *et al.*^61^ developed a methodology of detecting gas flaring activities and retrieving/calibrating gas flaring volumes from the visible band signal at night collected from the U.S. Air Force Defense Meteorological Satellite Program (DMSP) Operational Linescan System (OLS). Criteria of the best nighttime lights data for compositing require the absence of sunlight, moonlight, and clouds, and no contamination of solar glare and auroral emissions. The nighttime lights product used in the gas flaring analysis (called ‘lights index’) retrieved from DMSP is the average visible band digital number of cloud-free light detections multiplied by the percent frequency of light detection. The ‘sum of lights index’ is used to determine the magnitude of gas flaring. Gas flares are identified as ‘sum of lights index’ values of 8.0 or greater for all one km^2^ grid cell. Gas flares are further confirmed visually in the nighttime lights composites, including circular lighting features with a bright center and wide rims, the global population density grid, NASA MODIS satellite hot spot data, and Google Earth. Based on the sum of lights index values and reported gas flaring volumes for countries and individual flares, an algorithm has been developed to estimate gas flaring volumes^61^, i.e.,

(2)Volflaring=0.0000266×Sumoflightsindex,R2=0.976

Where Vol_*flaring*_ is the volume of gas flaring in the unit of BCM (billion cubic meters). The NOAA NGDC (National Geophysical Data Center) archives the long-term dataset of nighttime lights products, country-level gas flaring volumes and coverage areas (http://ngdc.noaa.gov/eog/download.html).

The nighttime lights products are available at the annual temporal resolution, which could account for the changes of long-term gas flaring activities. The coverage areas for all gas flaring source regions in the form of polygon shapefiles are time invariant. To distribute the global gas flaring BC emissions to any desired spatial resolutions, we follow the procedures as (1) Raster values from the nighttime lights products for a specific region are extracted according to its corresponding polygon shapefile, which constrains the gas flaring areas. All extracted rasters are kept at their original cell sizes (30-arc seconds). For certain years with single DMSP nighttime lights dataset, it is directly used for filtering out the rasters. For most years, two DMSP satellite datasets are available. For instance, the operation of F15 (the 15th DMSP satellite) and F16 overlapped from 2004–2007. In this case, we first take the average of the two datasets and then perform the same data processing method as the single DMSP dataset. (2) Rasters with values of less than 8 are excluded to preclude non-flaring activities. It is noted that the ECLIPSE dataset didn’t consider this step. (3) The screened ‘lights index’ data are further re-gridded from the original grid size of 30-arc seconds to a fine resolution of 0.1° × 0.1°. (4) Finally, the BC emission rates are calculated using the following equation:

(3)[Emii,j]c=[EFflare,BC]c×Volc×[Li,j]c/∑([Li,j]c)/Ai,j/T

where *c* represents a specific region, L represents the nighttime ‘lights index’ from gas flaring, and Vol_*c*_ represents the volume of gas flaring; In this study, Vol_*c*_ is based on Elvidge *et al.*’s^61^ DMSP time-series ending in the year 2012. Elvidge *et al.*^62^ have been using the Visible Infrared Imaging Radiometer Suite (VIIRS) on board the Suomi National Polar Partnership satellite for better detection of flares after 2012. Users can also choose other sources of flared volumes, e.g., the ATSR (Along Track Scanning Radiometer) time series from Casadio *et al.*^63^ or local data reports. A represents the area with grid resolution of 0.1°×0.1°; T is the total seconds in a year; [Emi_*i,j*_]_*c*_ represents the gas flaring BC emission rate (kg m^−2^ s^−1^) at grid cell [*i,j*] for the region *c*.

**Figure 2:**
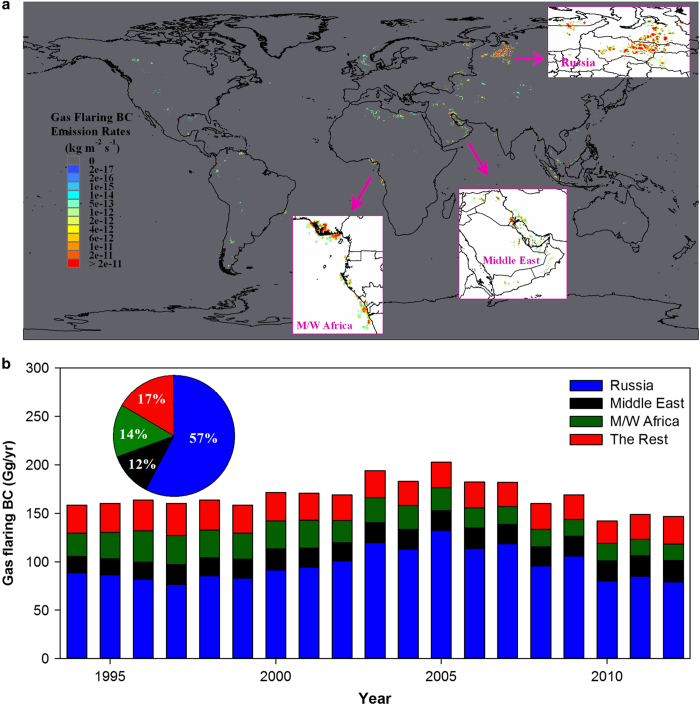
Gas flaring BC emissions at the grid and regional scale. (**a**) The spatial distribution of annual mean gas flaring BC emitting rates (unit: kg m^−2^ s^−1^) across the whole globe during 1994–2012 with subsets enlarging three hotspot regions: (1) Russia (mainly in the Khanty-Mansiysk and Yamalo-Nenets Autonomous Okrug) (2) the Middle East (including Iran, Iraq, Saudi Arabia, Qatar, Oman, Syria, United Arab Emirates, Kuwait, and Yemen) (3) the coastal areas of Middle and Western Africa (M/W Africa, including Nigeria, Angola, Gabon, Congo, Equatorial Guinea, Cameroon, and Democratic Republic of the Congo). (**b**) The annual gas flaring BC emissions (Gg/yr) of Russia, the Middle East, M/W Africa, and the rest of the world during 1994–2012. The pie chart inserted into **b** shows the average contributions from the defined four regions to the global total gas flaring BC emissions during 1994–2012.

Figure 2a shows the spatial distribution of annual mean gas flaring BC emission rates from 1994–2012 (Data Citation 1). The three strongest hotspot regions are enlarged in Fig. 2a, i.e., Russia, the Middle East, and the coastal areas of Middle and Western Africa (M/W Africa). Other hotspot regions with less flaring intensity include Northern Africa (e.g., Algeria, Libya, and Egypt), Southeast Asia (e.g., Indonesia and Malaysia), and Central Asia (e.g., Kazakhstan, Turkmenistan, and Uzbekistan). As for the rest of the gas flaring regions, BC emission intensities are relatively low.

Figure 2b shows the annual gas flaring BC emissions (Gg/yr) in Russia, the Middle East, M/W Africa, and the rest of the world from 1994–2012. Generally, the global gas flaring BC emissions vary relatively stable, ranging from around 160–170 Gg/yr except years from 2003 to 2007 exceeding 180 Gg/yr. Since 2005, there was a discernible decreasing trend in global gas flaring BC emissions, which is mainly attributed to the significant decrease (~40%) of gas flaring volumes in Russia. On the decadal scale, Russia dominates the global gas flaring BC emissions with an overwhelming fraction of about 57%, due to both its highest flaring volume and emission factor. The Middle East and M/W Africa contribute about 12 and 14%, respectively, while the rest of the world contributes around 17%.

## Data Records

### Data Record 1

The volumetric composition of associated petroleum gas with references and remarks, calculated fuel heating value, and EF_*flare,BC*_ (upper limit, lower limit and mean value) for each region are presented in the Excel format (Data Citation 1).

### Data Record 2

The global gas flaring BC emission rate products are provided in network Common Data Form (netCDF) format (Data Citation 1) with a ‘Geographic Lat/Lon’ projection and datum WGS-84. The dataset includes the following fields:

1. time: each year from 1994 to 2012;
2. lat: latitudes [−89.95°….89.95°] with interval of 0.1° for BC and area;
3. lon: longitudes [0.05°….359.95°] with interval of 0.1° for BC and area;
4. area: grid areas (unit: m^2^) Dimensions: [lat|1800]×[lon|3600]
5. BC: gas flaring BC emission rates for each year (unit: kg m^−2^ s^−1^) Dimensions: [time|19]×[lat|1800]×[lon|3600].

## Technical Validation

The validation of the data products is conducted by using the chemical transport modeling technique with available observation data. The Community Multi-scale Air Quality Model (CMAQ^64^) is used, which includes state-of-the-science capabilities for modeling multiple air quality issues. It is impossible to validate the gas flaring BC emissions in all the oil and gas production regions in this study, since non-flaring emissions often overwhelm the flaring emissions in most regions. By using chemical transport modeling as the data validation tool in this study, the uncertainty of dominant non-flaring emissions could potentially blur the outcome of the consideration of flaring emissions. For example, although the M/W Africa region is among the top three contributors to the world’s gas flaring BC emissions, it is in the region (NHAF: Northern Hemisphere Africa) where biomass burning activities are most intense in the world. According to the Global Fire Emissions Database (http://www.globalfiredata.org/analysis.html), the annual BC emissions from biomass burning in NHAF range from around 300 to 460 Gg/yr, a factor of 15–27 higher than the gas flaring emissions. Hence, it is crucial to select an ideal gas flaring region with minimal contributions from other BC emission sources. In this study, we have identified Russia, the Middle East, and the region downstream from Russia in the Arctic as the ideal regions for the data validation. In the following sections, we will explain the details.

To include most of the gas flaring source regions, we applied the hemispheric version of CMAQ (H-CMAQ), which encompasses the northern hemisphere with a Polar Stereographic projection^22,65^. The spatial horizontal resolution is set as 108 km×108 km with 180×180 grid cells and extends from the surface to 50 mb with 44 layers. CMAQv5.0.1 is configured with CB05 chemical mechanism and AER06 aerosol module. CMAQ is driven by the Weather Research and Forecasting (WRF) meteorology model version 3.5.1 with the same projection. As inputs for the WRF Preprocessing System (WPS), the National Centers for Environmental Prediction (NCEP) Final Analyses dataset (ds083.2) are used, with a resolution of 1.0°×1.0° for every six hours. A Meteorology/Chemistry Interface Processor (MCIP) 4.1 is used to link the WRF outputs to CMAQ.

We choose the year 2010 for demonstration, as this is the year that the most recent global anthropogenic emission inventory is available under the EDGAR (Emission Database for Global Atmospheric Research)-HTAPv2 (Hemispheric Transport of Air Pollution) project (http://edgar.jrc.ec.europa.eu/htap_v2/index.php?SECURE=123). EDGAR- HTAPv2 is a mosaic of regional and global emissions combing the latest available regional information with monthly temporal allocation and fine grid resolution of 0.1°×0.1°. It consists of aircraft, ship, energy, industry, transportation, residential, and agriculture sectors except for gas flaring. BC emissions from biomass burning are based on the Global Fire Emissions Database (GFEDv4s, http://www.globalfiredata.org/index.html).

### Validation against satellite AAOD in Russia’s gas flaring source regions

As the most substantial contributor to the global gas flaring BC emissions (Fig. 2b), Russia’s major flaring activities are in its Urals Federal District, where the human population is very sparse^22^. It was estimated that in the Urals, gas flaring dominated over its total BC emissions with a percentage of more than 90% (refs 17,22). In this regard, the Urals of Russia is an ideal region for its gas flaring emissions validation. Another advantage of choosing Russia could facilitate the assessment of the impact of gas flaring emissions in the Arctic region, where local emissions are minimal and more observation data is available.

**Figure 3:**
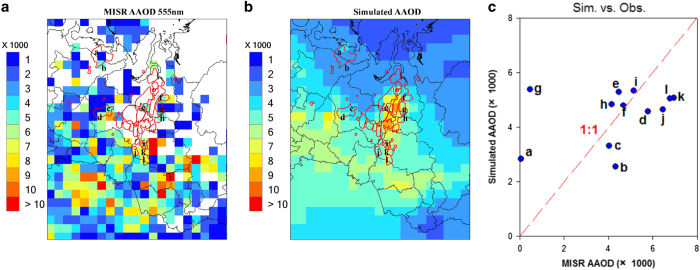
Comparison of AAOD between observation and simulation over Russia’s major flaring source region. (**a**) AAOD from MISR observation during the fall in 2010 (**b**) AAOD from H-CMAQ simulation during the same observation period. The gas flaring areas in Russia are denoted by the red polygons. The grids with valid AAOD values within the gas flaring areas are marked by alphabets from *a*−*i* in **a**. These grids are marked in **b** as well. (**c**) The scatter plot between MISR AAOD and simulated AAOD. Each scatter is marked by the alphabet corresponding to that shown in **a**,**b**. The 1:1 line is also plotted for reference.

It should be noted no ground-based measurements of ambient black carbon concentrations are available within or near this region. Alternatively, we choose the satellite aerosol products from MISR (The Multi-angle Imaging SpectroRadiometer) for the evaluation. AAOD (Absorption Aerosol Optical Depth) represents the columnar aerosol absorption and can be regarded as a suitable proxy of ambient particulate black carbon. Figure 3a,b compares the satellite-derived AAOD at 555nm from MISR and the CMAQ-simulated AAOD during the fall season in 2010. The reason to choose fall is that, on the one hand, almost no retrievals from satellite during the cold spring and winter are available in northern Russia (including the Urals), due to both difficulties of aerosol products retrievals over the high albedo surfaces with extended snow and ice cover during the cold seasons and the low angle of sun above the horizon. On the other hand, summer is not a suitable season, neither, as intense biomass burning occurs in Siberia which may interfere with the evaluation of gas flaring emissions. As shown in Fig. 3a, MISR still shows considerable missing values in the flaring areas (denoted by the red polygons) in the fall season. Grids with valid values within the gas flaring areas are extracted and denoted by alphabets from *a*−*i* in Fig. 3a. Corresponding values at the same locations (*a*−*i*) from CMAQ simulation (Fig. 3b) are also extracted, and their correlations are presented in Fig. 3c. It is shown that except at grids *a* & *g*, all other scatters lie relatively close to the 1:1 line. The values of AAOD over grids *a* & *g* observed from MISR are 1–2 magnitudes lower than the other investigated grids. It is expected that AAOD over the gas flaring emission source grids should be at a similar magnitude. In this regard, we could reasonably group grids *a* & *g* as outliers. By excluding these two grids from statistical analysis, the mean AAOD from MISR within the gas flaring areas is 5.3×10^−3^ and the corresponding mean AAOD from CMAQ simulation is 4.5×10^−3^. This results in a low NMB (Normalized Mean Bias) value of −14%, suggesting the estimated gas flaring BC emissions in Russia are reasonable.

### Validation against ground-based BC measurements in the Arctic

**Figure 4:**
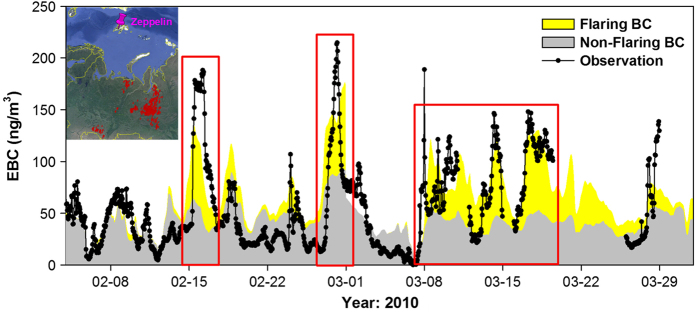
Comparison between hourly measurements of equivalent BC concentrations measured by a filter absorption photometer at the Zeppelin Observatory at Svalbard, Norway and simulated BC concentrations by H-CMAQ during February—March, 2010. The simulated BC concentrations are divided into flaring BC (yellow filled areas) and non-flaring BC (gray filled areas) concentrations by conducting brute-force simulation of zeroing out flaring emissions. The high observed BC episodes are highlighted by red rectangles. The inner plot visualizes the locations of gas flaring activities (red placemark) in the main oil and gas production fields in Russia (Khanty-Mansiysk and Yamalo-Nenets Autonomous Okrug) and the location of the Zeppelin Observatory (pink placemark).

Stohl *et al.*^17^ found that the role of gas flaring on the Arctic BC was underestimated based on the ECLIPSE estimates. Inclusion of gas flaring BC emissions into a Lagrangian particle dispersion model demonstrated obvious improvement in both magnitude and seasonality of simulated BC at ground-based Arctic observational sites. However, considerable discrepancy between observation and simulation was still found, especially during the high-BC episodes (see Fig. 9 in Stohl *et al.*^17^). In this regard, we revisit the role of gas flaring emissions on Arctic BC according to our new estimates of gas flaring BC emissions. Hourly measurements of equivalent BC concentrations measured by a filter absorption photometer at the Zeppelin Observatory at Svalbard, Norway (http://www.nilu.no/Miljoovervakning/tabid/186/language/en-GB/Default.aspx) are used for validating Russia’s gas flaring BC emission. We select February and March for investigation, as the winter-spring season is the so-called ‘Arctic Haze’ period that is characterized by the highest concentrations of air pollutants throughout the year^66,67^. As shown in Fig. 4, without gas flaring, the simulated BC time series (gray-filled areas) are relatively flat with hourly values mostly below 50 ng m^−3^. Compared to the observation (black dotted line), the strong temporal variation of BC concentrations fails to be reproduced, and the simulation misses almost all of the episodic BC peaks as highlighted in Fig. 4. Overall, on a monthly basis, the simulation without gas flaring emissions strongly underestimates the observed BC concentrations by 33% and 44% in February and March, respectively. By accounting for gas flaring emissions, the simulated BC concentrations from gas flaring (yellow-filled areas) stack on the simulated non-flaring BC concentrations, especially during the high BC episodes. Almost all the high BC peaks could be successfully captured, although variable discrepancies between the observation and simulation still exist. The wet removal efficiency of BC^68,69^ during the long-range transport, the unaccounted process that releases BC particles from condensed phases back to the interstitial air^70^, local emissions around the receptor site^71^, and of course the uncertainty of Russia’s gas flaring BC emissions may all account for those discrepancies. Overall, NMB values are reduced to −2% and 15% for February and March, respectively, suggesting a significantly improved model performance by including gas flaring BC emissions.

### Validation against satellite AAOD over the Persian Gulf in the Middle East

**Figure 5:**
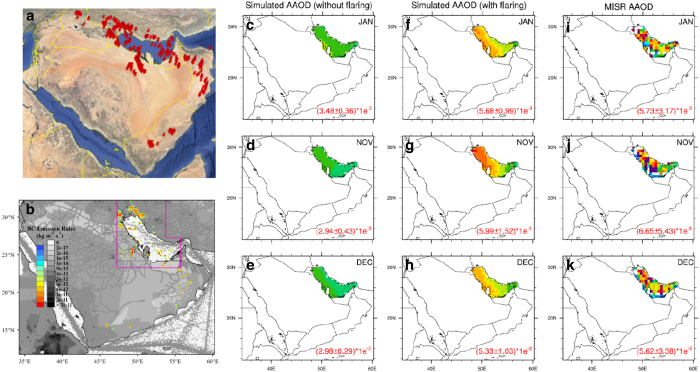
Comparison of AAOD between observation and simulation over the Persian Gulf. (**a**) Visualization of gas flaring activities in the Middle East in Google Earth (**b**) BC emission rates (kg m^−2^ s^−1^) from gas flaring (rainbow contour) and non-flaring emissions sectors (white/black contour, including energy/industry/traffic/residential/shipping sectors) based on the HTAPv2 dataset (**c**–**e**) Simulated AAOD without gas flaring emissions over the Persian Gulf in January, November, and December of 2010, respectively (**f**–**h**) The same as (**c**–**e**) but for simulated AAOD with gas flaring emissions (**i**–**k**) AAOD from MISR satellite observation.

As discussed earlier, the Middle East is also among the top three contributors to the global gas flaring BC emissions. Figure 5a visualizes the gas flaring hot spots detected from the DMSP satellites in the region of Middle East. It could be seen that gas flaring activities are mainly distributed along the coastline of the Persian Gulf. Also, it could be seen that there is a considerable number of flares over the gulf due to the operation of offshore oilfields there. Figure 5b overlays the BC emission rate from gas flaring on that from the non-flaring emission sectors based on the HTAPv2 dataset. As for the Persian Gulf and its surrounding areas (defined by the pink polygon in Fig. 5b), it is calculated that BC emissions from gas flaring are more than twice that of those from the non-flaring emission sectors. Hence, the dominance of gas flaring in this area facilitates the evaluation of gas flaring emissions by using numerical simulation. It should be noted that, similar to the Ural Federal District in Russia, ground-based observations are not available for the Persian Gulf region, neither. Thus, we also use AAOD from MISR for the evaluation. It should be also noted that as the Middle East is mostly arid and semi-arid, dust aerosol is a crucial component in the total particles over this region^72^. As AAOD retrieved from satellite is sensitive to both black carbon and dust, we do not evaluate the simulated AAOD compared to the satellite observations over the land, as the CMAQ model is not configured with dust simulation. Instead, we focus on the Persian Gulf. On the one hand, the gulf is an ocean surface and thus could eliminate the interference from direct dust emissions. On the other hand, most gas flaring activities cluster around and over the gulf. In this regard, the Persian Gulf is an ideal region for evaluating the gas flaring BC emissions in the Middle East.

Figure 5c–k show the comparisons between simulated and observed AAOD by masking regions except for the Persian Gulf. Figure 5c–e represent the spatial distribution of monthly mean AAOD simulated with non-gas flaring emissions only, while the simulated AAOD by accounting for gas flaring emissions are shown in Fig. 5f–h. Observed AAOD from MISR are shown in Fig. 5i–k for comparison. Only results for January, November, and December are presented, as these months are the lowest dust periods in the Middle East^73^. During the active dust season (i.e., summer and autumn), considerable fractions of AAOD are attributed to dust, which significantly interferes with the evaluation of BC emissions. In this regard, these months are not considered.

As we compare Fig. 5c–e,i–k, the simulated AAOD over the Persian Gulf show almost across-the-board low biases compared to observations if gas flaring emissions are not considered. The average AAOD values (with one standard deviation) over the Persian Gulf are calculated and shown in the right bottom corner of each plot. It is found that negative biases of 40–56% exist for the simulated AAOD without accounting for the gas flaring emissions. However, with the addition of gas flaring emissions, the simulated AAOD in all the investigated months are significantly enhanced. Overall, on a domain average, the impact of gas flaring on AAOD over the Persian Gulf could reach around 40–50%, suggesting gas flaring emissions could have been an overlooked source of aerosol absorption in the Middle East. Compared to observations (Fig. 5f–h vs i–k), the simulation could explain over 90% of the observed values with regard to the domain average, indicating satisfactory model performance and good representativeness of the emission dataset generated in this study. However, it is worth noting that the satellite observations show stronger spatial heterogeneity than the simulation. The less consistence in the spatial distribution between simulation and observation is possibly ascribed to several factors. Compared to the simulation that has no missing values for all grids in the domain, satellite observation often has no retrievals over certain grids, due to limited coverage of satellite swath and meteorological conditions such as clouds and precipitation. Secondly, although the investigated months we select are the low dust periods, dust is non-negligible in the atmosphere throughout the whole year in the Middle East. In the winter and spring seasons, the dominant anticyclone over the Arabian Peninsula facilitates the transport of mineral dust from deserts in the Arabian Peninsula and the Iranian Plateau to a widespread region in the Middle East, including the Persian Gulf. Hence, the interference of dust on AAOD retrieval from satellite is another cause. Finally, the omission of BC emissions from diesel engines for application in the oil and gas exploration, production, and transportation (e.g., powering pumps, drilling facilities, and other equipments, onsite electricity generation, and shipping vessels) could be another potential factor.

## Usage Notes

This dataset is an addition to the existing global black carbon emission inventories. Raw data can be reprocessed to any spatial resolution with changed projection (e.g., Lambert conformal conic, polar, etc) as inputs for air quality and climate models. This dataset can be utilized for assessing the black carbon pollutants level and long-term climate warming effects caused by black carbon.

## Additional Information

**How to cite**: Huang, K. & Fu, J. S. A global gas flaring black carbon emission rate dataset from 1994 to 2012. *Sci. Data* 3:160104 doi: 10.1038/sdata.2016.104 (2016).

**Publisher’s note**: Springer Nature remains neutral with regard to jurisdictional claims in published maps and institutional affiliations.

## Supplementary Material
